# Sensory Competition in the Face Processing Areas of the Human Brain

**DOI:** 10.1371/journal.pone.0024450

**Published:** 2011-09-02

**Authors:** Krisztina Nagy, Mark W. Greenlee, Gyula Kovács

**Affiliations:** 1 Institute of Psychology, University of Regensburg, Regensburg, Germany; 2 Department of Cognitive Science, Budapest University of Technology and Economics, Budapest, Hungary; University of Leuven, Belgium

## Abstract

The concurrent presentation of multiple stimuli in the visual field may trigger mutually suppressive interactions throughout the ventral visual stream. While several studies have been performed on sensory competition effects among non-face stimuli relatively little is known about the interactions in the human brain for multiple face stimuli. In the present study we analyzed the neuronal basis of sensory competition in an event-related functional magnetic resonance imaging (fMRI) study using multiple face stimuli. We varied the ratio of faces and phase-noise images within a composite display with a constant number of peripheral stimuli, thereby manipulating the competitive interactions between faces. For contralaterally presented stimuli we observed strong competition effects in the fusiform face area (FFA) bilaterally and in the right lateral occipital area (LOC), but not in the occipital face area (OFA), suggesting their different roles in sensory competition. When we increased the spatial distance among pairs of faces the magnitude of suppressive interactions was reduced in the FFA. Surprisingly, the magnitude of competition depended on the visual hemifield of the stimuli: ipsilateral stimulation reduced the competition effects somewhat in the right LOC while it increased them in the left LOC. This suggests a left hemifield dominance of sensory competition. Our results support the sensory competition theory in the processing of multiple faces and suggests that sensory competition occurs in several cortical areas in both cerebral hemispheres.

## Introduction

In everyday life we are typically exposed to multiple stimuli within our visual field simultaneously. For example, a person’s face is usually surrounded by objects and another faces as well, such as when a person is in a crowd. Still, there are relatively few available studies on the neural processing of simultaneously presented multiple stimuli compared to studies that are focussed on a single isolated stimulus. We know from previous studies that multiple stimuli presented within the visual field compete for neural representations in the visual cortex [1] (for a review see [2]). Theories of sensory competition suggest that the processing capacity of multiple simultaneously presented stimuli within the receptive field of a given neuron is limited, presumably due to the mutual suppressive interactions among them. Signs of such interactions have been found in several areas of both the ventral and dorsal visual pathways, using extracellular single-cell recording techniques in the macaque brain [3]–[8]. More recently, human functional magnetic resonance imaging (fMRI) studies also confirmed the results of single-cell recording experiments and showed the existence of competitive interactions among multiple stimuli in the human visual cortex [2], [9]–[15]. In these studies stimuli were either presented sequentially or simultaneously. The lower blood-oxygen-level-dependent (BOLD) signal in the simultaneous condition was interpreted as a sign of competitive interactions among stimuli. Such interactions have been found in V1, V2, V4 and TEO of the human visual cortex.

However, most of the above studies used simple or more complex shapes and objects as stimuli. Thus, while several studies have been performed on the sensory competition effects among non-face stimuli relatively less is known about the interactions in the human brain for multiple face stimuli. The scarcity of fMRI data regarding competition of face stimuli is surprising, since it has been suggested previously that the face processing system has its own, face-specific attentional system and its own capacity limits [16]–[18]. Limited capacity of resources necessary for face identification or recognition [19]–[22] as well as for gender discrimination [23] has already been demonstrated behaviourally. Jacques and Rossion [24]–[26] used event related potential (ERP) recordings to study the neural correlates of multiple face stimuli. They found that if a distractor face was presented peripherally to the central target stimulus, than the amplitude of the face-sensitive, occipito-temporal N170 component was reduced in comparison with the condition when the distractor was a phase-randomized noise image. In another study, similar signs of competition were found on the N170 ERP component for inverted faces as well [27]. Agam et al. [28] recorded intracranial field potentials in the human visual cortex and found a small attenuation of the response, when the preferred stimulus was paired with a non-preferred one, supporting the existence of competitive interactions in face processing.

Up to now only a few neuroimaging studies are available on the competition among faces. The effects of clutter and diverted attention on the category selective information of the fusiform face area (FFA) and parahippocampal place area (PPA) were studied by Reddy and Kanwisher [29]. They found that information about the preferred categories of an area is robust to the clutter of the visual stimulation, created by a face and house image presented on either side of the fixation spot. Similar stimulus arrangements were used in a subsequent study to test how responses to simultaneous stimuli are combined and how these responses are affected by attention in these areas using multivoxel pattern analysis [30]. They found that the response to a pair of stimuli could be described by the average of their individual responses in the multidimensional voxel space, supporting the theory of biased-competition [31]. Gentile and Jansma [32] used pairs of more or less similar faces to test the theory of biased competition as a suitable model of stimulus selection [31]. They found lower BOLD signal in the FFA when the two simultaneously presented (and task- irrelevant) faces were similar than when they were dissimilar. This suggests that similar stimuli, encoded by overlapping neuronal populations compete more with each other than dissimilar ones do. Axelrod and Yovel [33] applied composite stimuli, containing preferred and related, but non-preferred stimuli of the FFA (faces and glasses). If a preferred (face) and a non-preferred (glasses) stimulus were presented next to each other significant release from adaptation was found, suggesting that the FFA is sensitive to the non-preferred stimulus on the preferred one and that there is an interaction between the two.

These previous neuroimaging studies (with the exception of [32]) have tested the effect of other stimulus categories (houses and various objects) on the processing of faces, presenting a face and an object along each other. However, theories of biased competition [34] as well as current neuroimaging studies [32] provided evidence that the competition effect is most pronounced for similar stimuli, belonging to the same category and exciting overlapping neuronal populations.

Another common property of most of the previous neuroimaging studies of competition is that they usually present stimuli alone or in pairs with component stimuli on opposite sides of the central fixation spot. This means that the two stimuli are presented in opposite hemifields. However, studies of object [35], scene [36] and face processing [37] suggest that contralateral and ipsilateral stimuli are processed differentially and object [38] and face processing [39] are, to a large extent, position-dependent. In addition, a previous study, using checkerboard patterns, proved that contralateral peripheral stimulation is reduced by competition with ipsilateral stimulation only in inferior temporal cortex [40].

Therefore, we designed an experiment to test the sensory competition among stimuli falling into the same category (faces) within the same visual hemifield. Importantly, another deviation from the previous studies was that we presented always the same number of visual stimuli, but varied the ratio of stimuli preferred by the face sensitive areas (i.e. faces) systematically. We presented the faces together with phase-randomized noise images, as it has been shown by Jacques and Rossion [24] that the magnitude of early ERP components is reduced when a lateralized face is presented in the context of another face, compared to a situation when it is presented together with a phase-randomized noise image. We reasoned that the competition related reduction in BOLD signal in face processing areas would be higher in situations when faces are intermixed with phase-randomized noise images than when a single face is presented together with noise images. Previous studies showed that the degree of competitive interactions changes as a function of the spatial separation of the competing stimuli in the array: the larger the spatial separation among the stimuli, the lower the magnitude of competitive interactions (for review see [2]). Thus we varied the number of interleaving noise images (consequently the distance) among the face stimuli, expecting the largest reduction of the BOLD signal with the smallest spatial separation. Both of the prior hypotheses were confirmed by our results regarding the FFA and the lateral occipital complex (LOC), but not the occipital face area (OFA), supporting the notion that sensory competition exists among neurons that process face stimuli. However, sensory competition has a varying effect on face processing areas of the ventral system.

## Results

### Behavioral control experiment

The stimuli of the present experiments are presented on the periphery, thus, it is important to prove that it is possible to solve the famous face detection task without eye-movements, by fixating the central fixation spot. Further, the difficulty of detecting a famous face among other faces (for example in our 4F condition) might be more difficult compared to the condition where a single face is presented among non-face stimuli (1F condition). In addition, top-down effects, such as selective spatial attention can also modulate sensory competition [2]. Therefore, we performed a behavioral experiment outside the scanner to compare performance, reaction times and eye-movements across the conditions having different ratio of face stimuli.

**Figure 1:**
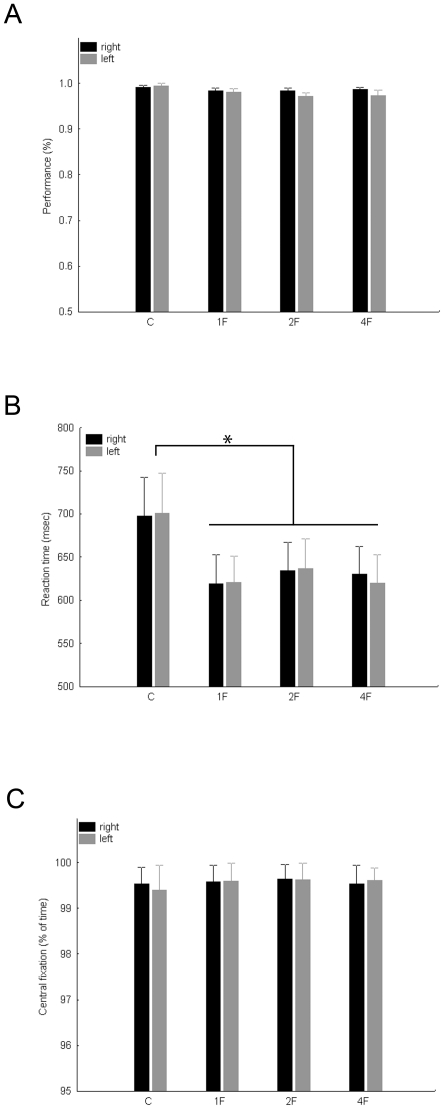
Subjects’ performance, reaction times and fixation performance in the behavioral control experiment separately for the Control (C), 1F, 2F (averaged across 2F0G, 2F1G and 2F2G conditions) and 4F conditions and for right (black) and left (gray) visual fields. A. Average detection performance. B. Average reaction times of the celebrity detection task. C. Eye-fixation performance expressed as proportion of trial-time (see Methods for details). Error bars represent standard error of means.

Fig. 1A shows the average performance of celebrity detection for the different conditions, separately for the left and right visual hemifields. Neither the main effect of hemifield (F(1, 7) = 1.26, p>0.15), nor the main effect of condition (F(1, 7) = 1.26, p>0.15) nor the interaction between the two factors (F(3, 21) = 1.06, p>0.3) were significant. Moreover, while the reaction time in the C condition, which contains no faces at all, was significantly longer than in the other conditions (Fig. 1B, main effect of condition: F(3, 21) = 4.26, p<0.02) we observed no differences in reaction time among the 1F, 2F and 4F conditions (post-hoc tests: p>0.5 for each comparison). Neither the main effect of hemifield (F(1, 7) = 0.01, p>0.8), nor its interaction with stimulus condition was significant (F(3, 21) = 1.07, p>0.3) for the reaction times.

We expressed the fixation performance of our subjects as the proportion of time during a trial, spent within the 2 deg circle around the fixation spot. As Fig. 1C suggests subjects could fixate during the task very well and importantly their fixation performance was not different for the experimental conditions (main effect of hemisphere: F(1, 7) = 0.1, p>0.7, main effect of condition: F(3, 21) = 1.42, p>0.2, interaction: F(3, 21) = 0.28, p>0.8).

Altogether, these results suggest that task difficulty, response strategy and fixation performance are comparable among the different conditions and could not explain any difference of the BOLD response in the fMRI experiment.

### Face selective regions

#### Fusiform Face Area

**Figure 2:**
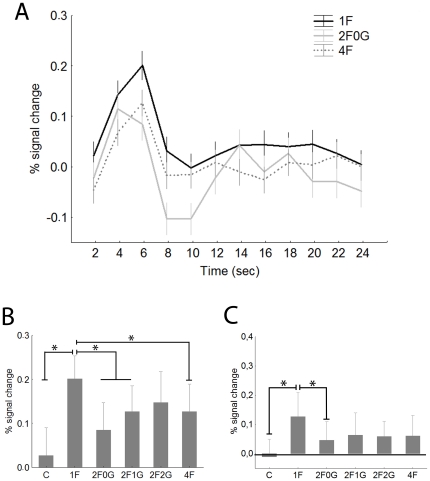
The right FFA. A Time course (mean ±standard error) of fMRI activity for contralateral visual presentations. For sake of clarity only the 1F (black), 2F0G (gray) and 4F (dashed) conditions are depicted. Data derived from a finite impulse response (FIR) model with 2 s time bins. B. Average peak (4−6 sec) activation profiles (±standard error) of the right FFA for the six experimental conditions for contralateral stimulation. C. Average peak activation profiles (±standard error) of the right FFA for ipsilateral stimulation. C- control, noise images, 1F-single face, 2F0G- two neighbouring face images and two noise images, 2F1G- two faces with one noise image in between them, 2F2G- two faces, separated by two noise images, 4F- four face images. * - Fishers post-hoc comparisons: p<0.05.

**Figure 3:**
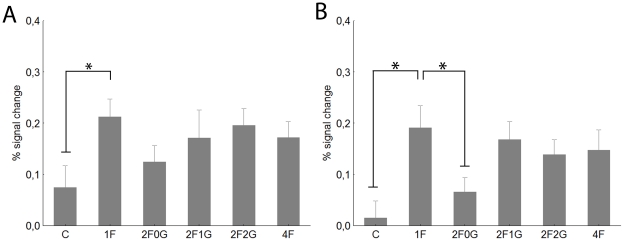
Average peak activation profiles (±standard error) of the left FFA. A. Contralateral stimulation. B. Ipsilateral stimulation. For conventions see Fig. 2.

Fig 2A presents the typical hemodynamic response functions of the right FFA (rFFA) for the 1F, 2F0G and 4F conditions. The rFFA showed significantly more pronounced responses when a face was presented among the noise images than the control stimulus, composed of four noise images for both contralateral (i.e. left visual hemifield, Fig. 2B) and ipsilateral stimulation (Fig 2C, paired t-test for single face condition (1F) versus the control (C) condition: t = 4.6, p<0.0001 and t = 2.8, p<0.01 for contra- and ipsilateral stimuli, respectively). The response pattern of the left FFA (lFFA, Fig. 3) was similar to that of the rFFA. The 1F condition led to significantly higher responses than the Control conditions both for the contralateral (i.e. right visual hemifield, Fig 3A) and ipsilateral (i.e. left visual hemifield, Fig. 3B) stimulation (t-tests: t = 2.6, p<0.01 and t = 3.4, p<0.01 for contra- and ipsilateral comparisons, respectively).

Since we found a significant main effect of hemisphere (three-way ANOVA with hemisphere, hemifield and conditions as within subject factors -see Methods section: F(1,40) = 4.5, p<0.04) on the BOLD signal of the FFA (in addition to the main effects of visual hemifield (F(1,40) = 17.8, p<0.001) and conditions (F(4,160) = 4.3, p<0.005)) in the following we present our data for the right and left FFA separately.

For the rFFA the BOLD signal was significantly different for the stimuli with different ratio of faces (main effect of condition in a two-way within subject ANOVA with hemifields and conditions as factors- see Methods section: F(4, 160) = 3.6; p<0.01) and for the two hemifields (main effect of hemifield: F(1, 40) = 14.0; p<0.001). As is obvious from Fig. 2, the largest response was evoked in the 1F condition while the two-face condition with no gap (2F0G, for detailed description please see Methods), the two-face condition with one noise stimulus in between (2F1G) and the four-face condition (4F) evoked significantly lower responses (Fisher post-hoc tests: p<0.01 for each comparison). This suggests that if more faces are presented to the visual system the response in the rFFA is reduced when compared to a single face presentation, possibly due to the competition among the face stimuli. Our results also suggest that such competition among faces has a negative correlation with the inter-stimulus distance for the contralateral stimuli (Fig. 2B): while we could observe strong reduction of the BOLD signal in both the 2F0G and 2F1G conditions the 2F2G condition, where the two face images were separated by two noise images, did not lead to a significantly lower signal than the 1F condition (Fischer post hoc tests against 1F condition: p<0.001, p<0.04 and p = 0. 4 for 2F0G, 2F1G and 2F2G conditions, respectively). This conclusion is supported further by the results of a separate one-way within-subject ANOVA, performed separately on the three contralateral 2F conditions: we observed a significant main effect of stimulus distance (F(2,80) = 4.1, p<0.03) and a significantly larger response in the 2F2G condition when compared to the 2F0G (Fisher post-hoc test: p<0.01).

Although the interaction of visual hemifield and condition was not significant (F(4,160) = 0.6, p = 0.6) the response pattern was somewhat different for the ipsilateral visual hemifield (Fig. 2C). The various two-face conditions and the 4F condition led to similar response magnitudes and only the 2F0G condition was different from the single face condition (Fishers post-hoc test: p<0.05). Furthermore, there were no differences among the various 2F and 4F conditions (Fisher post-hoc tests for each comparisons: p>0.6), suggesting similar response reductions. This is supported further by the separate one-way ANOVA for the ipsilateral 2F conditions where we have not observed significant differences (F(2,80) = 0.1, p = 0.9).

**Figure 4:**
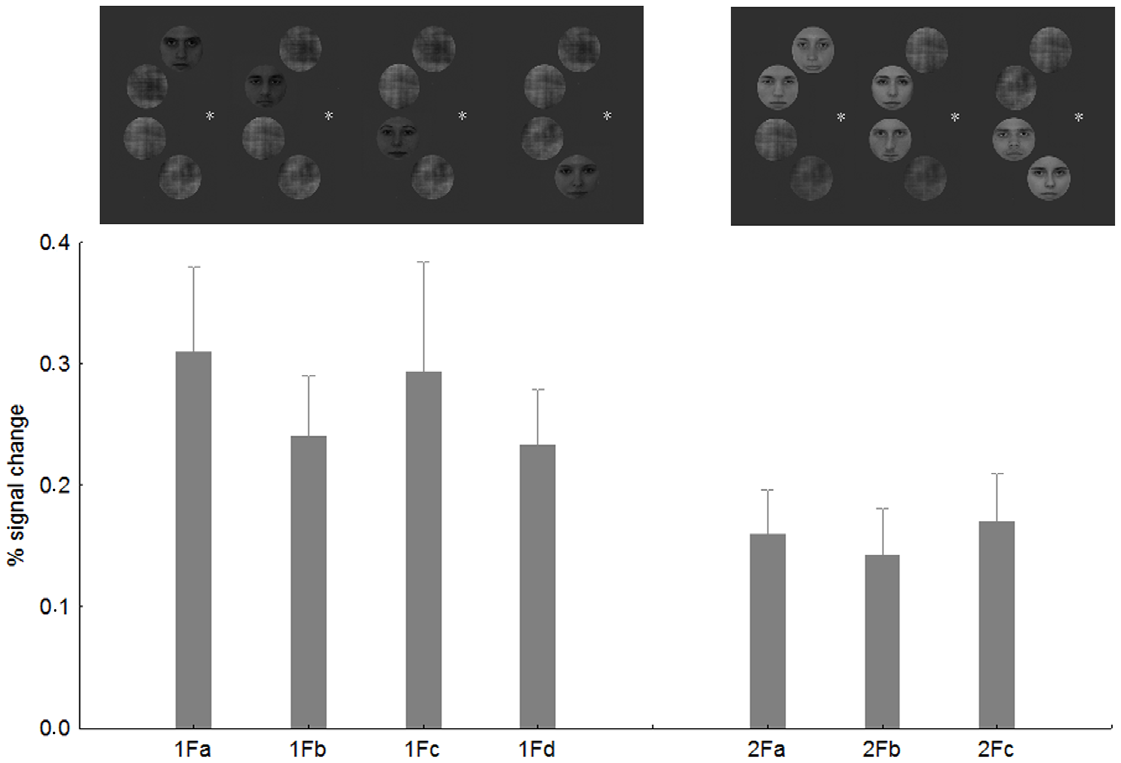
The average BOLD signal from the rFFA in the four possible 1F conditions and in the three possible 2F0G conditions. Insets show examples of the stimuli.

Is the observed competition effect specific to certain parts of the visual field? To test this question we performed an additional analysis. First, we split the contralateral 1F conditions into four separate regressors: 1F_a_- face appearing in the uppermost position, 1F_b_- face in the second position from above, 1F_c_- face in the third position from above and 1F_d_- face on the bottom (see Fig. 4 for examples). Second, we split the contralateral 2F0G conditions into three groups: 2F0G_a_-the two neighboring faces were in the two upper positions, 2F0G_b_-two faces in the middle, 2F0G_c_-two faces in the two lower positions. Next we extracted the BOLD signal for these 14 conditions separately from the rFFA, using the same coordinates and ROI size as before.

Fig. 4 shows the results of the position specificity analysis. The average of the four 1F conditions, although due to the lower number of repetitions per condition were more noisy than in the previous model, led to significantly larger BOLD signal than that of the 2F0G conditions (t-test for dependent samples: t = 3.28, p<0.002), confirming the previous analysis and suggesting competing interactions among face stimuli in rFFA. One-way within subject ANOVA performed on the 1F condition with four levels revealed no significant differences among the four possible positions of the face stimuli (F(3,195) = 0.3, p>0.8). Similarly, one-way ANOVA performed on the 2F0G condition (3 within-subject levels) also failed to find any differences among the stimulus positions (F(2,130) = 0.13, p>0.8). Altogether, these results suggest that the absolute position of the faces within the hemifield is not important in determining sensory competition effects of the rFFA. To the contrary, the results are similar across different positions within the contralateral visual field.

The response reduction of the lFFA (Fig. 3) was similar to that of the rFFA: increasing the number of simultaneously presented face stimuli led to a significant signal reduction (main effect of condition: F(4, 160) = 3.5, p<0.01). Neither the main effect of hemifield (F(4, 40) = 0.1, p = 0.15), nor its interaction with stimulus condition (F(4, 160) = 0.01, p<0.8) was significant but post-hoc test for the 1F vs 2F0G comparison suggest that the reduction is somewhat stronger for the ipsilateral (p<0.005) than for contralateral stimuli (p<0.11).

#### Occipital Face Area

Since we found a significant main effect of visual hemifield (F(1,40) = 38.6, p<0.0001) and a hemifield x hemisphere interaction (F(1,40) = 8.6, p<0.005) for OFA, we present our data separately for the two sides and hemifields.

**Figure 5:**
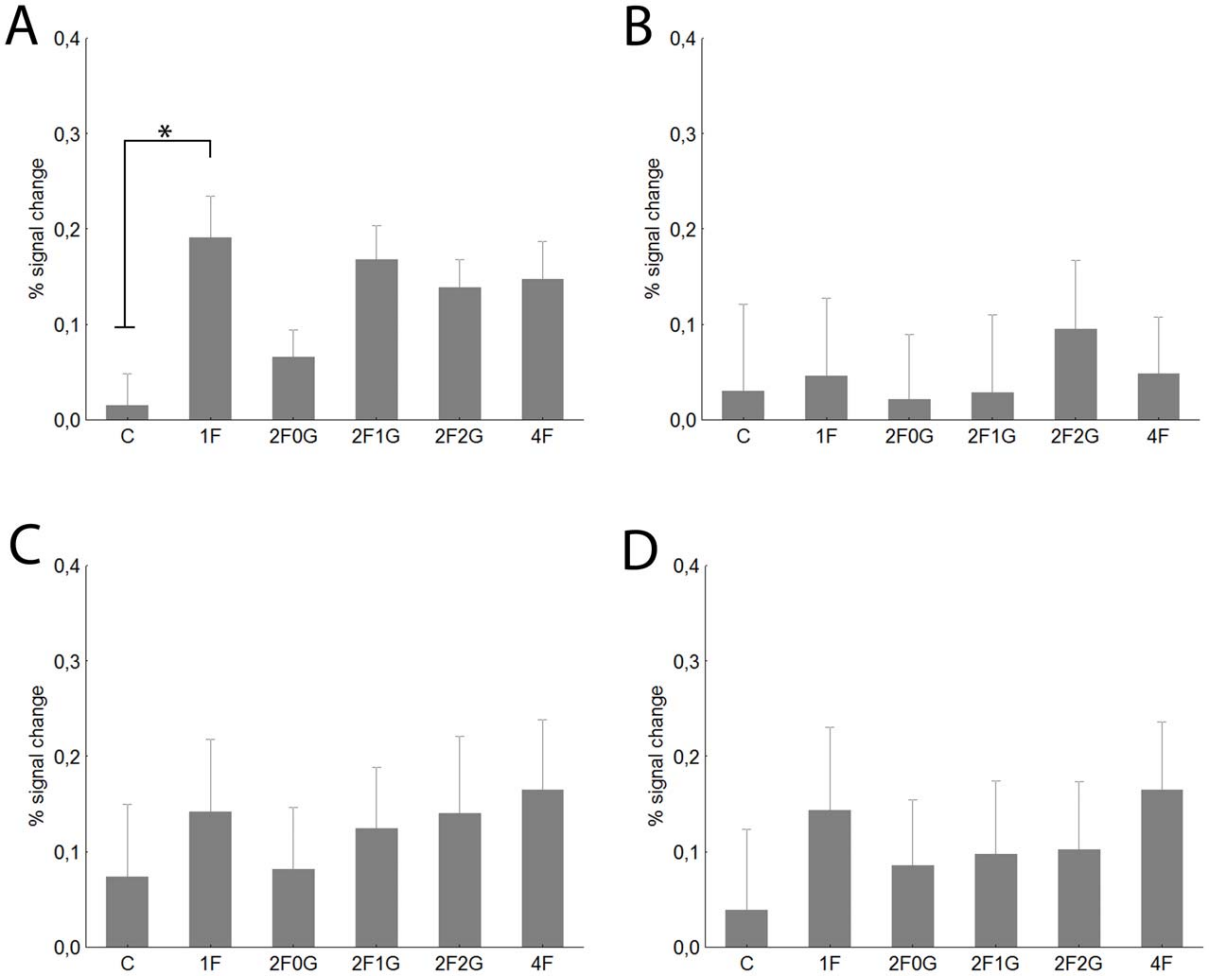
Average peak activation profiles (±standard error) of the OFA. Contralateral (rOFA: A, lOFA: C) and ipsilateral (rOFA: B, lOFA: D) stimulation. For conventions see Fig. 2

For contralateral stimuli the 1F condition evoked significantly higher response magnitudes than did the Control condition (t-test: t = 2.7, p<0.01) in the right OFA (rOFA) (Fig. 5A). However, as previous studies (showing that this area has receptive fields more biased towards the contralateral hemifield than those in FFA [41]) would suggest, the ipsilateral response (Fig. 5B) was not at all different between these two conditions (t-test for 1F vs C: t = 0.3, p>0.8). For the left OFA neither ipsi- nor contralateral stimuli led to different responses in the C and 1F conditions (Fig. 5C and D, t-tests for C vs 1F: t<1.3, p>0.1 for both hemifields).

Furthermore, we did not observe any difference in the activity of right and left OFA as a function of the number of faces present (main effect of condition: F(4,160) = 0.83, p = 0.5 and F(4,40) = 1.9, p = 0.12 for the right and left OFA, respectively; interaction of condition and hemifield: F(4,160) = 0.87, p = 0.4 and (F(4,160) = 0.2, p = 0.9 for the right and left OFA, respectively). The only comparison showing a tendency for response suppression was between the contralateral 1F and 2F0G conditions of the rOFA (Fishers post-hoc test: p = 0.09). This suggests that the OFA and its major target area the FFA have considerably different sensory competition properties.

### Object selective regions: the Lateral Occipital Complex

**Figure 6:**
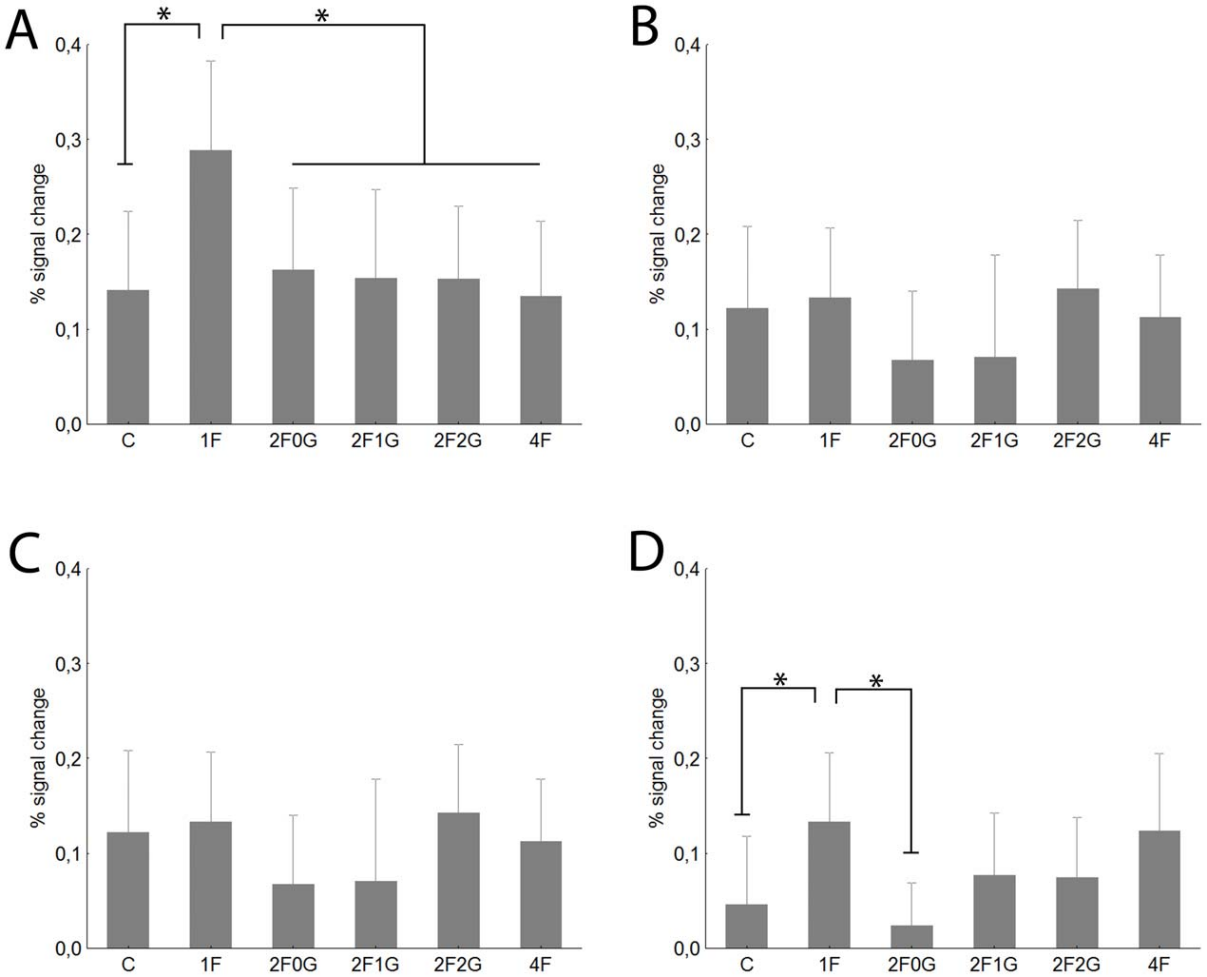
Average peak activation profiles (±standard error) of the LOC. Right LOC contralateral (A) and ipsilateral stimulation (B). Left LOC contralateral (C) and ipsilateral (D) stimulation. For conventions see Fig. 2.

To test the specificity of any possible competition effect to the face-processing network we determined the location of the lateral occipital cortex (LOC) of our subjects as well. For contralateral stimulation (Fig. 6A) we observed significantly stronger responses in the 1F than in the C condition (t-test: t = 2.7, p<0.01) supporting the object specificity of the right LOC (rLOC). Similarly to what we found for rOFA, ipsilateral stimulation (Fig. 6B) led to no significant differences between C and 1F (t-test for C vs 1F: t = 1.15, p>0.2) for rLOC either. While contralateral stimulation led to similar responses in C and 1F condition for the left LOC (lLOC) (Fig. 6C, t-test for C vs 1F: t<0.4, p>0.7) the 1F condition led to higher responses than C for ipsilateral stimulation (Fig. 6D, t-test: t = 2.2, p<0.03).

Since the analysis revealed a main effect of hemisphere (F(1,40) = 3.8, p<0.05) and visual hemifield (F(1,40) = 17.6, p<0.001), as well as the interaction of hemisphere with stimulus condition (F(4,160) = 2.7, p<0.05) in the following we present our data for the left and right LOC separately.

To our surprise, we observed a very strong reduction of the response in the rLOC when presenting two or four faces simultaneously (main effect of condition: F(4, 160) = 2.7, p<0.05) and this effect was superseded by a significant visual hemifield effect (F(1,40) = 12.4, p<0.01), due to larger responses in the contra- than ipsilateral hemifield. As post-hoc tests for the comparisons of 1F with two or four faces suggest the single face condition evoked larger BOLD signal than any other condition for the contralateral stimulation (post-hoc tests for the comparisons of 1F with every other condition: p<0.005) but not for the ipsilateral stimulation (p>0.18 for each comparison). This supports previous studies [41] pointing to the existence of contralaterally biased receptive fields in LOC and suggests different competition properties in the two hemifields.

For the left LOC (lLOC) we only observed a small, but significant main effect of condition (F(4, 160) = 2.5, p<0.04) which was due to the smaller BOLD signal in the 2F0G than in the 1F condition during ipsilateral stimulation (Fig. 6D, post-hoc test for 1F vs 2F0G: p<0.005). No other comparison was significant. This surprising result suggests that lLOC, in spite of the contralaterally biased receptive fields, is able to code some information in the ipsilateral hemifield as well.

**Figure 7:**
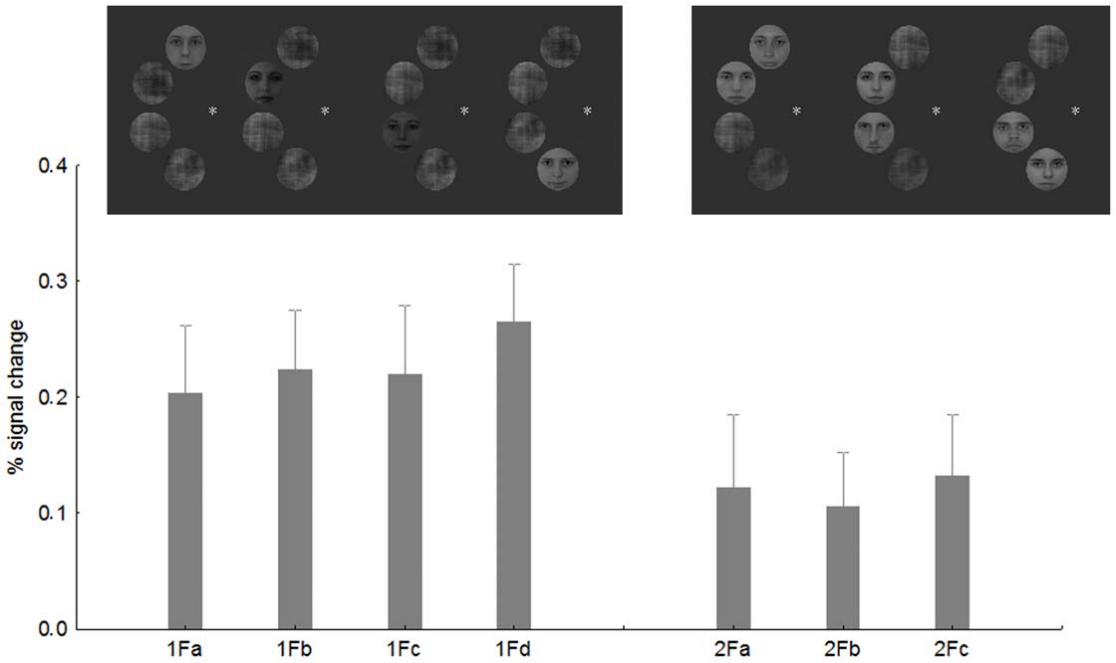
The results of the position analysis for rLOC. For conventions see Fig. 2 and Fig. 3.

As for rFFA, we have also tested for LOC if the observed competition effect is specific to an absolute location within the visual field or not (see above for details of methods of this analysis). Fig. 7 shows the results of the position specificity analysis. The average of the four contralateral 1F conditions led to significantly larger BOLD signal than that of the 2F0G conditions (t-test for dependent samples: t = 2.56, p<0.01), replicating the results of the previous analysis and suggesting competing interactions among face stimuli in rLOC. One-way within-subject ANOVA performed on the 1F condition revealed no significant differences among the four positions of the face stimuli (F(3,120) = 0.27, p>0.8). Similarly, one-way ANOVA performed on the contralateral 2F0G condition showed no differences among the stimulus positions (F(2,80) = 0.08, p>0.9). Overall, these results suggest that the specific position of the faces is not important in determining sensory competition effects in the rLOC. Similarly to what was found for rFFA and rLOC, one-way within subject ANOVA performed on the 1F condition revealed no significant differences among the four ipsilateral positions of the face stimuli (F(3,243) = 0.4, p>0.7) or among the three ipsilateral 2F0G condition (F(2,162) = 0.53, p>0.5) within the lLOC.

### Early visual cortex (EVC)

**Figure 8:**
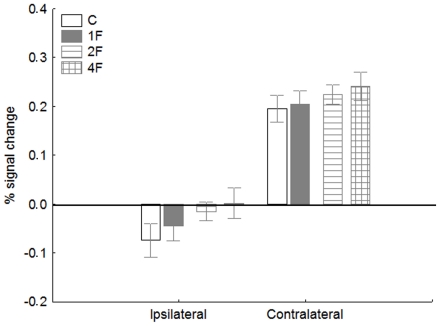
Average peak activation profiles (±standard error) of the early visual cortex (EVC, averaged across hemispheres) for the ipsilateral and contralateral stimulations. For conventions see Fig. 2

To test if the observed competition effects are specific to the occipito-temporal visual areas we extracted the hemodynamic response functions of the EVC for the left and right hemispheres along the calcarine sulcus, using the Fourier noise vs. faces+objects contrast of the functional localizer scans. Fig. 8 shows the peak BOLD signals from EVC (averaged over the two hemispheres) for contralateral and ispilateral stimulations. A within-subject ANOVA with ipsi- and contralateral stimulation and stimulus conditions (4: C, 1F, 2F (0, 1 and 2G conditions are collapsed for this analysis), 4F)) as factors shows that there is no difference in BOLD signal as a function of ratio of face stimuli in EVC (main effect of stimulus condition: F(3, 204) = 2.0, p>0.1 and interaction of stimulus position and condition: F(3, 204) = 0.17, p>0.9). This result suggests that the observed competition effect in FFA and LOC is not due to any low-level variation of the stimulus.

Moreover, the fact that we observed enhancement in the BOLD signal for contralateral but not for ipsilateral stimulation (main effect of stimulus position: F(1, 68) = 91.3, p<0.00001)) corresponds to the literature [42] and also suggests that our subjects fixated properly during the scanning and systematic differential fixation performance cannot explain the observed competition effects.

### Whole brain analysis

Finally, we also performed a whole-brain random-effects analysis for 4F > 1F, 4F > 2F (0, 1 and 2G conditions are collapsed for this analysis) as well as for the 1F > 4F and 2F > 4F contrasts for left and right visual hemifield stimuli separately. Such comparisons, in addition to test whether other areas reflect the sensory competition processes as well, test also if attention was equally allocated across the different conditions. If subjects were not attending or fixating in the various conditions similarly than that would have shown up in the whole brain statistical maps (specially at the peripheral representations of the EVC). However, neither of the above contrasts led to significant activations in additional brain regions, even at the liberal threshold of Puncorrected< 0.0001, suggesting similar attentional processes, fixation performance across conditions and emphasizing the role of the occipito-temporal areas in sensory competition.

## Discussion

Our major results are the following: (1) Increasing the ratio of faces in a composite display, containing the same number of stimuli reduces the BOLD signal bilaterally in FFA and in the rLOC; (2) Increasing the distance among faces reduces the competitive interactions in FFA; (3) The magnitude of competition depends on the visual hemifield of the stimuli: ipsilateral stimulation reduces the competition effects in the rLOC and increases it in the LOC of the left hemisphere.

Theories of sensory competition suggest that simultaneously presented stimuli compete for neural representation and this mutual suppressive interaction is manifest both on the single-cell and population levels. In neuroimaging experiments competition is usually tested by one of two ways. In one set of paradigms stimuli are either presented sequentially alone or simultaneously together while in the other type single stimulus presentation is compared with pairs of stimuli. In such paradigms usually lower BOLD signal [32], [33] and reduced information in the multivoxel pattern [30] was observed in the simultaneous conditions in various areas of the occipito-temporal cortex. In our present experiments we introduce a novel way to test competitive interaction. We chose to keep the absolute number of stimuli constant and to manipulate the ratio of faces. Both ERP [24] and neuroimaging studies [10], [32] suggest that stimulus similarity modulates competitive interactions in the ventral visual pathway. Since (1) the occipito-temporal cortex usually responds more vigorously to faces than to the phase-scrambled noise images and (2) the sensory competition, expressed as the amplitude reduction of the N170 ERP component is larger among faces than between face and noise images [24] we compared the competition among faces with the competition between face and phase-randomised noise images. Keeping the absolute number of stimuli constant gave us a chance to study the nature of face-face and face-noise image competition independent of the number of stimuli presented.

Biased competition theories of attention state that the ongoing competition among stimuli is biased by attention in a way that if attention is directed towards one of the multiple stimuli, the mutually competitive effects are reduced [6], [7], [11]. One important aspect of our current data is that to ensure that attention was paid equally to all faces we required subjects to perform a recognition task with respect to the faces and we only analyzed the non-target trials. A possible effect of this task is that subject did not attend to the phase-noise images, which could increase the competitive interactions among neurons coding them as well as with those for the face stimuli. This could lead to lower BOLD signal for the C condition. The fact, however, that we observed the largest BOLD signal in the 1F condition (where the possible suppression effect of the noise images is largest on the face evoked response) both in rFFA and rLOC argues against this conclusion. However, undoubtedly further studies are necessary to test how attention and other top-down effects interact with the observed sensory competition effects of the present study. Such studies have already been published regarding perceptual grouping and illusory contours [15].

The fact that we observed lower BOLD signal in the left and right FFA is in agreement with previously mentioned fMRI results. Gentile and Jansma [32] found that two similar, but task irrelevant faces, presented in opposite hemifields competed with each other more and led to lower BOLD signals in the FFA than dissimilar faces. Our results suggest that phase-randomized stimuli that lead to lower activations in the higher extrastriate areas of the occipito-temporal cortex in human and monkeys [43]–[48] and lead to lower face sensitive ERP component amplitudes [49], [50] compete with a face less than do two faces among each other. This competition, however, is different along the ventral processing pathway. While competition led to lower BOLD signal both in the FFA and rLOC we observed no such effect in the OFA. Previously the OFA was shown to be involved in early processing of facial features in fMRI, transcranial magnetic stimulation experiments and in lesion studies of acquired prosopagnosia [51]–[55]. Furthermore, studies of brain-damaged prosopagnosic patients emphasize the independent role of OFA and FFA [56] in face processing. In the study of Gentile and Jansma [32] an area, having very similar coordinates (40, −75, −3) to those of the rOFA of the current study showed competition effects. Methodological differences may be responsible for the different results. While we compared multiple faces against a face presented together with phase-randomised noise images Gentile and Jansma [32] compared pairs of similar and dissimilar faces. Their method, presumably being more specific to neurons that encode small differences between faces, might have detected smaller changes that the face vs. noise context comparisons of the present study could overlook. The tendency in right OFA of 1F and 2F0G conditions being different (p = 0.07) in the present study supports this argument.

Area LOC is preferentially activated by complex object shapes (for reviews see [57]–[58]) including faces [59]. In our experiments right LOC showed strong BOLD signal reduction in every condition where more than one face was present. This suggests that the observed competition effects are not face-specific, but occur in such areas where the preferred stimulus is not a face at all. Previous studies of competition, using objects as stimuli, found that responses to object pairs within LOC were well predicted by the averages of responses to their constituent objects, suggesting competition between stimuli for the limited neural bandwidths. Our results show that this competition in LOC is not limited to objects, but appears also for face stimuli. These results together would suggest a differential role of LOC and OFA in processing multiple face stimuli. It is worth noting that ipsilateral stimulation, while evoking activation in FFA and (more surprisingly) in the right LOC reduced competition among stimuli. This finding suggests that multiple stimuli are processed differentially in the two hemifields, a conclusion we discuss further below.

Recent fMRI [38] and single-cell [60] studies suggest the position specificity of object selective areas. The LOC and another object selective area along the posterior part of the fusiform sulcus (pFS) were shown to process information in a position-constrained manner [38] (but see [61] for another conclusion regarding LOC, using multivariate pattern classification). Contrary to this we have not observed any differences of the BOLD signal or of the competition effect across the specific positions of our composite stimuli. This apparent disagreement could be explained by differences of the stimulation (single line-drawings of objects vs. varying proportion of faces/noise images) and analysis techniques (iterative split-half correlation analysis vs. ROI based analysis of the BOLD signal). Furthermore, we always presented the stimuli within a hemifield, where the effect of position is weaker when compared to that of between hemifields [38]. Thus, it is possible that the less sensitive ROI approach overlooks the relatively smaller position specificity within a given hemifield. Nevertheless, the issue of position specificity in human object-selective cortical areas remains unresolved and is currently under intense debate.

Previous studies, using colorful complex patterns, showed that the degree of competitive interactions changes as a function of the spatial separation of the competing stimuli in the array: the larger the spatial separation among the stimuli, the smaller the magnitude of competitive interactions (for a review see [2]). This effect is the most prominent in earlier visual areas V2 and V4, suggesting that the effect of spatial separation is the strongest where the neuronal receptive fields are small and it is not present at all in primate area TEO, an area located on the fusiform gyrus, medial and superior to right FFA, where the receptive fields are larger than 7 deg in diameter. Our results regarding the right FFA show a different pattern. We observed that increasing the spatial separation of competing faces reduced the competition effect (i.e. led to larger BOLD signals) in right FFA. Whether the different stimuli, the slightly different coordinates or the fact that we varied the distance by varying the number of phase-noise images, the finding that separating the faces led to the different results requires further investigation.

A surprising result of the current work regards the inter-hemifield and inter-hemispheric differences of the competition effects. It seems that the LOC shows larger magnitude of competition effects in the left hemifield of both hemispheres. So far no direct comparison has been made regarding the receptive field sizes of LOC and FFA. Nevertheless, several lines of evidence suggest that neurons of LOC retain more location information when compared to FFA [62]-[64]. Further, there is evidence of a larger contralateral stimulus preference in LOC and OFA than in FFA [35], [41]. Thus, in case of LOC, the significant ipsilateral responses indicate the existence of neurons with receptive fields, centered in the ipsilateral hemifield, similarly to studies of face [37] and object adaptation [36].

However, the observed competition effect is not entirely identical in FFA and LOC: while we observed a significant distance effect (i.e. less competition for more distant stimuli) for left hemifield stimuli both in right FFA and left FFA no such effect was observed in the right LOC. This suggests that the spatial extent of competitive interactions is smaller in FFA than in LOC, a conclusion requiring further proof. Together these results raise the possibility that the previously observed left hemifield advantage of face perception is the result of an efficient interhemispheric integration at higher levels [65], [66].

It has been previously suggested that the face processing system has its own, face-specific attentional system and its own capacity limits [16]–[18]. Limited capacity of resources necessary for face identification or recognition [19]–[22] as well as for gender discrimination [23] have already been demonstrated. The reduced activity in rFFA for multiple face stimuli serves as a possible neural correlate of such an effect. Nevertheless, the exact nature of the relationship between selective attention, multiple face representation and sensory competition will undoubtedly require further studies.

## Materials and Methods

### Subjects

Eight subjects (mean age: 29.3 yrs, 3 females) participated in the behavioural control experiment. Twelve healthy university students participated in the fMRI experiment (mean age: 26 yrs, SD: 3.3 yrs). Seven of them were female, one left-handed. All subjects had normal or corrected-to normal vision and they provided their written consent in accordance with the protocols approved by the Ethical Committee of the University of Regensburg.

### Stimulation and Procedure

**Figure 9:**
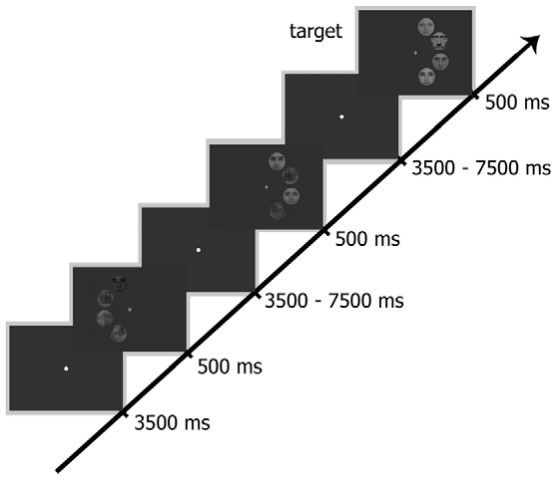
Procedures and example stimuli. Timeline depicts the stimuli of 1F and 2F1G conditions in the left and right visual hemifield, respectively as well as an example of a target trial (4F, right hemifield).

Faces were gray-scale, full-front digital photos of eight female and male faces, chosen from a large pool of photos, partially overlapping the stimulus database of Kovács et al [37], [67]. Faces (mean luminance: 18 cd/m^2^) had no obvious gender-specific features, such as facial hair, jewellery or make-up. They were fit behind an oval mask (radius = 3.5 deg), eliminating outer contours of the faces. The Fourier phase-randomized versions of the faces were created by the algorithm of Nasanen [68]. Next, we constructed stimuli having four equidistant positions on a semicircle on the right or on the left side of the fixation spot (radius = 4 deg, distance between individual stimuli: 0.7 deg). These four positions were occupied by four noise images (Control, C) one face and three noise images (1F), two faces and two noise images or four faces (4F), positioned randomly at the four locations. We also manipulated the inter-stimulus distance between face stimuli in conditions where two faces and two noise images were presented: the two face stimuli could occupy neighbouring positions (no gap between the faces- 2F0G) or they could be separated by one (2F1G) or by two noise images (2F2G). The position of the face and noise images was chosen randomly for each trial in the 1F and 2F conditions, but for the main analysis we collapsed our data across the various positions. Thus altogether we had six stimulus conditions in the left and six in the right visual field. For stimulus examples see Fig. 9.

Stimuli were back-projected via an LCD video projector (JVC, DLA-G20, Yokohama, Japan, 72 Hz, 800×600 resolution) onto a translucent circular screen (app. 30° deg diameter), placed inside the scanner bore at 63 cm from the observer. Stimulus presentation was controlled via Matlab (The Mathworks, Natick, MA), using PsychToolbox 2.45 [69].

Stimuli were presented for 500 ms and were followed by an ITI of either 3500 or 7500 ms, randomly (Fig. 9). Subjects were required to fixate a centrally presented fixation spot, which was present throughout the entire trial. To ensure that covert attention was paid equally to all objects, we required subjects to count silently the number of stimulus occurrences where a previously chosen familiar celebrity (either a portrait of Hugh Laurie or Marcia Cross) occurred on a randomly selected position. Such target trials (mean occurrence: 7 trials/block, mean detection performance: 97% ± 2.5%) could be in any of the five conditions, containing at least one face. In the subsequent analysis only non-target trials are included. During one run (approximately 16 min long) we presented 91 trials with different number of faces in the left or right visual hemifield randomly. Participants were first familiarised with the stimuli and the task lasted for approximately 5 min. Subsequently, they carried out four runs during the experiment.

### Parameters and Data Analysis

Imaging was performed using a 3-Tesla MR Head scanner (Siemens Allegra, Erlangen, Germany). For the functional series we continuously acquired images (29 slices, 10 deg tilted relative to axial, T2* weighted EPI sequence, TR  = 2000 ms; TE  =  30 ms; flip angle  =  90 deg; 64×64 matrices; in-plane resolution: 3×3 mm; slice thickness: 3 mm). High-resolution saggital T1-weighted images were acquired using a magnetization EPI sequence (MP-RAGE; TR  =  2250 ms; TE  =  2.6 ms; 1 mm isotropic voxel size) to obtain a 3D structural scan.

Details of preprocessing and statistical analysis are given elsewhere [37], [70]. Briefly, the functional images were corrected for acquisition delay, realigned, normalized to the MNI-152 space, resampled to 2×2×2 mm resolution and spatially smoothed with a Gaussian kernel of 8 mm FWHM (SPM8, Welcome Department of Imaging Neuroscience, London, UK).

Regions of interests (ROI) analysis was based on the results of separate functional localiser runs (488 sec long, 17 s epochs of faces, objects and their Fourier randomised versions (size: 6 deg, central presentation) interleaved with 17 s of blank periods, 2 Hz; 300 ms exposition time; 200 ms blank, MARSBAR 0.42 toolbox for SPM [71].

The location of face responsive areas was determined individually as areas responding more strongly to faces than to objects and to Fourier noise images in the functional localiser scans (p_uncorrected_ <0.0001; T = 7.53, FFA [average Talairach coordinates (±SE): 42(4), −56(7), −19(4) and −43(6), −61(8), −20(5) for left the and right hemispheres, respectively]) and the OFA [average Talairach coordinates (±SE): 40(5), −81(6), −13(3) and −39(5), −84(9), −11(6) for left and right hemispheres]). Areas selectively responding to objects were determined by similar functional localiser scans comparing the activity for nonsense objects vs. their Fourier randomised versions and faces (p_uncorrected_<0.0001; T = 7.53, area LOC: [average Talairach coordinates (±SE): 51(5), −73(6), −4(5) and −47(8), −73(10), −1(6) for left and right hemispheres]).

The portion of the early visual cortex (EVC), responding to the stimulation of the central 6 deg visual field was determined by the Fourier noise vs. faces+objects contrast (p_uncorrected_<0.0001; T = 7.53, [average Talairach coordinates (±SE): 8(4), −91(3), −6(4) and −6(3), −89(6), 8 (6) for the left and right hemispheres]). This location corresponded to the projection zone for that eccentric visual-field location along the calcarine sulcus of both hemispheres.

The ROIs were selected individually on the single subject level from these thresholded T-maps. Areas matching our anatomical criteria and lying closest to the corresponding reference cluster (resulting from the random-effects analysis for differential contrasts; p_uncorrected_ <0.0001; T = 7.53) were considered as their appropriate equivalents on the single subject level. A time series of the mean voxel value within an 8 mm radius sphere around the local peak of the areas of interest was calculated and extracted from our event-related sessions using finite impulse response (FIR) models [72]. The convolution of a reference hemodynamic response function with box-cars, representing the onsets and durations of the experimental conditions, was used to define the regressors for a general linear model analysis of the data.

Six different event types were analysed and modelled at the onset of test stimuli. The peak of the event-related averages in a window from 4 to 6 s was used as an estimate of the magnitude of the response and was averaged across observers. Analysis was performed in three steps. First, to test the difference between the C and 1F condition we performed a simple paired t-test for each ROI, hemisphere and visual field separately. Second, to test the effect of hemisphere we performed a large, three-way ANOVA with hemisphere (2), hemifield (2) and stimulus condition (6 levels: C, 1F, 2F0G, 2F1G, 2F2G, 4F) for each FFA, OFA and LOC separately. Finally, to determine the effect of the number of faces the percent signal change values were compared by two-way repeated measured ANOVAS with visual hemifield (2) and stimulus condition (5 levels: 1F, 2F0G, 2F1G, 2F2G, 4F) as factors. Post-hoc analysis was performed by Fisher LSD tests.

### Behavioral control experiment

Stimuli, stimulus size, conditions and trial structure were identical to those of the fMRI experiment. The only exception was that, in order to be able to measure reaction times, subjects performed a two alternative forced-choice celebrity detection task. After each trial they had to decide if a celebrity was presented on the display or not by pressing a button on a keyboard. Subjects were tested in a dimly lit room (average background luminance <1 cd/m^2^). Stimuli were presented on a 17“ monitor (1024×768 pixel resolution, 75 Hz vertical refresh rate with a viewing distance of 63 cm) on a uniform grey background. Subjects were asked to fixate a central fixation spot and their eye-movements were controlled by an infrared eye-tracking system (IView X RED, SMI, Germany). The proportion of trial-time, spent in a 2 deg vicinity of the fixation spot was calculated off-line for each subject, visual hemifield and condition separately and was used as a measure of fixation performance. Detection performance and reaction time were averaged for each condition, hemifield and subject separately and a within-subject two-way ANOVA was performed with hemifield (2) and experimental conditions (4: C, 1F, 2F (2F0G, 2F1G and 2F2G conditions were collapsed for this analysis), 4F) as factors.

## References

[1] Duncan J, Inui T, McClelland JL (1996). Cooperating brain systems in selective perception and action.. Attention and performance XVI.

[2] Beck DM, Kastner S (2008). Top-down and bottom-up mechanisms in biasing competition in the human brain.. Vision Research.

[3] Britten KH, Heuer HW (1999). Spatial summation in the receptive fields of MT neurons.. Journal of Neuroscience.

[4] Miller EK, Gochin PM, Gross CG (1993). Suppression of visual responses of neurons in inferior temporal cortex of the awake macaque by addition of a second stimulus.. Brain Research.

[5] Missal M, Vogels R, Orban GA (1997). Responses of macaque inferior temporal neurons to overlapping shapes.. Cerebral Cortex.

[6] Recanzone GH, Wurtz RH, Schwartz U (1997). Responses of MT and MST neurons to one and two moving objects in the receptive field.. Journal of Neurophysiology.

[7] Reynolds JH, Chelazzi L, Desimone R (1999). Competitive mechanisms subserve attention in macaque areas V2 and V4.. Journal of Neuroscience.

[8] Snowden RJ, Treue S, Erickson RG, Andersen RA (1991). The response of area MT and V1 neurons to transparent motion.. Journal of Neuroscience.

[9] Beck DM, Kastner S (2005). Stimulus context modulates competition in human extrastriate cortex.. Nature Neuroscience.

[10] Beck DM, Kastner S (2007). Stimulus similarity modulates competitive interactions in human visual cortex.. Journal of Vision.

[11] Kastner S, De Weerd P, Desimone R, Ungerleider LG (1998). Mechanisms of directed attention in the human extrastriate cortex as revealed by functional MRI.. Science.

[12] Kastner S, De Weerd P, Pinsk MA, Elizondo MI, Desimone R (2001). Modulation of sensory suppression: Implications for receptive fields sizes in the human visual cortex.. Journal of Neurophysiology.

[13] Macevoy SP, Epstein RA (2009). Decoding the representation of multiple simultaneous objects in human occipitotemporal cortex.. Current Biology.

[14] McMains SA, Kastner S (2010). Defining the units of competition: influences of perceptual organization on competitive interactions in human visual cortex.. Journal of Cognitive Neuroscience.

[15] McMains SA, Kastner S (2011). Interactions of top-down and bottom-up mechanisms in human visual cortex.. Journal of Neuroscience.

[16] Neumann MF, Schweinberger SR, Wiese H, Burton AM (2007). Event-related potential correlates of repetition priming for ignored faces.. Neuroreport.

[17] Neumann MF, Schweinberger SR (2009). N250r ERP repetition effects from distractor faces when attending to another face under load: Evidence for a face attention resource.. Brain Research.

[18] Neumann MF, Mohamed TN, Schweinberger SR (2011). Face and object encoding under perceptual load: ERP evidence.. Neuroimage.

[19] Bindemann M, Jenkins R, Burton AM (2007). A bottleneck in face identification: repetition priming from flanker images.. Experimental Psychology.

[20] Boutet I, Chaudhuri A (2001). Multistability of overlapped face stimuli is dependent upon orientation.. Perception.

[21] Jenkins R, Lavie N, Driver J (2003). Ignoring famous faces: category-specific dilution of distractor interference.. Perception and Psychophysics.

[22] Palermo R, Rhodes G (2002). The influence of divided attention on holistic face perception.. Cognition.

[23] Bindemann M, Burton AM, Jenkins R (2005). Capacity limits for face processing.. Cognition.

[24] Jacques C, Rossion B (2004). Concurrent processing reveals competition between visual representations of faces.. Neuroreport.

[25] Jacques C, Rossion B (2006). The time course of visual competition to the presentation of centrally fixated faces.. Journal of Vision.

[26] Jacques C, Rossion B (2007). Electrophysiological evidence for temporal dissociation between spatial attention and sensory competition during human face processing.. Cerebral Cortex.

[27] Sadeh B, Yovel G (2010). Why is the N170 enhanced for inverted faces? An ERP competition experiment.. Neuroimage.

[28] Agam Y, Liu H, Papanastassiou A, Buia C, Golby AJ (2010). Robust selectivity to two-object images in human visual cortex.. Current Biology.

[29] Reddy L, Kanwisher N (2007). Category selectivity in the ventral visual pathway confers robustness to clutter and diverted attention.. Current Biology.

[30] Reddy L, Kanwisher NG, VanRullen R (2009). Attention and biased competition in multi-voxel object representations.. Proceeding of the National Academy of Sciences of the United States of America.

[31] Desimone R, Duncan J (1995). Neural mechanisms of selective visual attention.. Annual review of neuroscience.

[32] Gentile F, Jansma BM (2010). Neural competition through visual similarity in face selection.. Brain Research.

[33] Axelrod V, Yovel G (2011). Nonpreferred stimuli modify the representation of faces in the fusiform face area.. Journal of Cognitive Neuroscience.

[34] Reynolds JH, Desimone R (1999). The Role of Neural Mechanisms of Attention in Solving the Binding Problem.. Neuron.

[35] McKyton A, Zohary E (2007). Beyond retinotopic mapping: the spatial representation of objects in the human lateral occipital complex.. Cerebral Cortex.

[36] MacEvoy SP, Epstein RA (2007). Position selectivity in scene- and object-responsive occipitotemporal regions.. Journal of Neurophysiology.

[37] Kovács G, Cziráki C, Vidnyánszky Z, Schweinberger SR, Greenlee MW (2008). Position-specific and position-invariant face aftereffects reflect the adaptation of different cortical areas.. Neuroimage.

[38] Kravitz DJ, Kriegeskorte N, Baker CI (2010). High-level visual object representations are constrained by position.. Cerebral Cortex.

[39] Afraz A, Pashkam MV, Cavanagh P (2010). Spatial heterogeneity in the perception of face and form attributes.. Current Biology,.

[40] Schwartz S, Vuilleumier P, Hutton C, Maravita A, Dolan RJ (2005). Attentional load and sensory competition in human vision: modulation of fMRI responses by load at fixation during task-irrelevant stimulation in the peripheral visual field.. Cerebral Cortex.

[41] Hemond CC, Kanwisher NG, Op de Beeck HP (2007). A preference for contralateral stimuli in human object- and face-selective cortex.. PLoS One,.

[42] Tootell RB, Hadjikhani N, Hall EK, Marrett S, Vanduffel W (1998). The retinotopy of visual spatial attention.. Neuron,.

[43] Epstein R, Kanwisher N (1998). A cortical representation of the local visual environment.. Nature.

[44] Grill-Spector K, Kushnir T, Edelman S, Itzchak Y, Malach R (1998). Cue-invariant activation in object-related areas of the human occipital lobe.. Neuron.

[45] Kanwisher N, McDermott J, Chun MM (1997). The fusiform face area: a modul in human extrastriate cortex specialized for face perception.. Journal of Neuroscience.

[46] Kourtzi Z, Kanwisher N (2000). Cortical regions involved in perceiving object shape.. Journal of Neuroscience.

[47] Rainer G, Augath M, Trinath T, Logothetis NK (2002). The effect of image scrambling on visual cortical BOLD activity in the anesthetized monkey.. Neuroimage.

[48] Sawamura H, Georgieva S, Vogels R, Vanduffel W, Orban GA (2005). Using functional magnetic resonance imaging to assess adaptation and size invariances of shape processing by humans and monkeys.. Journal of Neuroscience.

[49] Jemel B, Schuller AM, Cheref-Khan Y, Goffaux V, Crommelinck M (2003). Stepwise emergence of the face-sensitive N170 event-related potential component.. Neuroreport.

[50] Rossion B, Gauthier I, Tarr MJ, Despland P, Bruyer R (2000). The N170 occipito-temporal component is delayed and enhanced to inverted faces but not to inverted objects: and electrophysiological account of face-specific processes in the human brain.. Neuroreport.

[51] Gauthier I, Tarr MJ, Moylan J, Skudlarski P, Gore JC (2000). The fusiform “face area” is part of a network that processes faces at the individual level.. Journal of Cognitive Neuroscience.

[52] Pitcher D, Walsh V, Yovel G, Duchaine B (2007). TMS evidence for the involvement of the right occipital face area in early face processing.. Current Biology.

[53] Rossion B, Caldara R, Seghier M, Schuller AM, Lazeyras F (2003). A network of occipito-temporal face-sensitive areas besides the right middle fusiform gyrus is necessary for normal face processing.. Brain.

[54] Schiltz C, Rossion B (2006). Faces are represented holistically in the human occipito-temporal cortex.. Neuroimage.

[55] Sorger B, Goebel R, Schiltz C, Rossion B (2007). Understanding the functional neuroanatomy of acquired prosopagnosia.. Neuroimage.

[56] Rossion B (2008). Constraining the cortical face network by neuroimaging studies of acquired prosopagnosia.. Neuroimage.

[57] Grill-Spector K, Kourtzi Z, Kanwisher N (2001). The lateral occipital complex and its role in object recognition.. Vision Research.

[58] Malach R, Levy I, Hasson U (2002). The topography of high-order human object areas.. Trends in Cognitive Sciences.

[59] Lerner Y, Hendler T, Ben-Bashat D, Harel M, Malach R (2001). A hierarchical axis of object processing stages in the human visual cortex.. Cerebral Cortex.

[60] Goris RL, Op de Beeck HP (2009). Neural representations that support invariant object recognition.. Frontiers in Computational Neuroscience.

[61] Cichy RM, Chen Y, Haynes JD (2011). Encoding the identity and location of objects in human LOC.. Neuroimage.

[62] Schwarzlose RF, Swisher JD, Dang S, Kanwisher N (2008). The distribution of category and location information across object-selective regions in human visual cortex.. Proceeding of the National Academy of Sciences of the United States of America.

[63] Sayres R, Grill-Spector K (2008). Relating retinotopic and object-selective responses in human lateral occipital cortex.. Journal of Neurophysiology.

[64] Grill-Spector K, Dickinson S, Tarr M, Leonardis A, Schiele B (2009). What has fMRI taught us about object recognition?. Object Categorization: Computer and Human Vision Perspectives.

[65] Compton RJ (2002). Inter-hemispheric interaction facilitates face processing.. Neuropsychologia.

[66] Schweinberger SR, Baird LM, Blümler M, Kaufmann JM, Mohr B (2003). Interhemispheric cooperation for face recognition but not for affective facial expressions.. Neuropsychologia.

[67] Kovács G, Zimmer M, Bankó É, Harza I, Antal A (2006). Electrophysiological correlates of visual adaptation to faces and body parts in humans.. Cerebral Cortex.

[68] Nasanen R (1999). Spatial frequency bandwidth used in the recognition of facial images.. Vision Research.

[69] Brainard DH (1997). The psychophysics toolbox..

[70] Cziráki C, Greenlee MW, Kovács G (2010). Neural correlates of high-level adaptation-related aftereffects.. Journal of Neurophysiology.

[71] Brett M, Johnsrude IS, Owen AM (2002). The problem of functional localization in the human brain.. Nature Reviews Neuroscience.

[72] Ollinger JM, Schulman GL, Corbetta M (2001). Separating processes within a trial in event-related functional MRI.. Neuroimage.

